# Digital workflow for implant-supported restorations in the mandible including reverse scan bodies: a feasibility study

**DOI:** 10.1186/s12903-026-09507-9

**Published:** 2026-08-04

**Authors:** Aiste Kernen-Gintaute, M Akulauskas, F Kernen, S Wenger, NU Zitzmann, BC Spies, F Burkhardt

**Affiliations:** 1 Department of Prosthetic Dentistry, Center for Dental Medicine, Medical Center - University of Freiburg, Faculty of Medicine, University of Freiburg, Hugstetter Str. 55, Freiburg, 79106 Germany; 2 Department of Reconstructive Dentistry, University Center for Dental Medicine, University of Basel, Basel, Switzerland; 3 Department of Oral and Maxillofacial Surgery, Translational Implantology, Medical Center -University of Freiburg, Faculty of Medicine, University of Freiburg, Freiburg, Germany; 4 Biomedical Engineering Institute, Kaunas University of Technology, Kaunas, Lithuania

**Keywords:** Digital workflow, Intraoral scanner, Oral implants, Passive fit, Reverse scan bodies, Mandible

## Abstract

**Background:**

Completely edentulous mandibles present a challenge for accurate intraoral scanning, which can compromise the passive fit of implant-supported restorations. Reverse scan bodies may improve the accuracy of implant position transfer and support a fully digital workflow. However, clinical evidence for their use in the edentulous mandible remains limited.

**Methods:**

In this single-patient prospective clinical evaluation, one fully edentulous mandible restored with two interforaminal implants was scanned ten times using an intraoral scanner (IOS). Based on each scan, titanium verification bars (VBs) were fabricated, and their passive fit was assessed clinically. After sectioning and intraoral re-splinting, splinted verification bars (SVBs) were digitized using reverse scan bodies and a laboratory scanner to fabricate new verification bars (NVBs). Linear and angular deviations between datasets (IOS, VB, SVB, NVB) were analyzed using metrology software and statistically evaluated with Welch’s ANOVA and post hoc tests (α = 0.05).

**Results:**

All VBs and NVBs demonstrated passive fit clinically. Mean linear deviations were 12 μm between IOS and VB, 50 μm between VB and SVB (*p* = 0.001), and 26 μm between SVB and NVB (*p* = 0.20). Mean angular deviations were 0.18° between IOS and VB, with six measurements > 0.4°; 0.79° between VB and SVB (*p* = 0.01), with seven measurements > 0.4°; and 0.06° between SVB and NVB, with four measurements > 0.4°.

**Conclusions:**

Within the limitations of this single-patient evaluation and the favorable two-implant mandibular configuration, intraoral scanning provided sufficient accuracy for fabricating passively fitting restorations on two implants in the edentulous mandible. Measurable linear and angular deviations did not compromise clinical fit in this case. The use of reverse scan bodies may be effective in cases of misfit after intraoral re-splinting, especially in more complex full-arch workflows, as only minor linear and angular deviations were observed in the NVBs with integrated abutments.

## Background

Digital workflows are increasingly used for the impression, design, and fabrication of dental prostheses on both natural teeth and implants [1, 2]. Intraoral scanning offers a nearly contactless impression procedure that patients generally perceive as more comfortable compared with conventional impression techniques using elastomeric impression materials [3]. In addition, digital workflows enable a more time- and cost-efficient approach [4]. However, completely edentulous arches pose a particular challenge for digital impressions, as they provide fewer anatomical landmarks and reference points than dentate arches, which are essential for accurate stitching of intraoral images [5, 6].

Accurate recording of implant positions is essential for the long-term success of implant-supported restorations, as a passive fit can minimize both mechanical and biological complications [7, 8]. A misfit between the restoration and the implants has been associated with prosthetic component fractures, screw loosening, and peri-implant bone loss [7, 8]. Implant positions are typically captured by an intraoral scanner using scan bodies that are attached to the implants [9, 10]. These scan bodies serve as geometric reference markers, allowing the laboratory software to determine the exact implant position and orientation [11].

Single-unit implant restorations and short-span implant-supported fixed dental prostheses fabricated using intraoral scanning and digital manufacturing techniques have demonstrated clinically acceptable accuracy [12, 13]. However, in completely edentulous arches, deviations exceeding 100 μm and 0.4º have been reported, which has so far limited the recommendation of routine clinical use for this indication [14, 15]. To improve the accuracy of complete-arch implant scans, several strategies have been proposed, including splinting impression copings to create an optical reference framework [16, 17]. Nevertheless, despite these modifications, the literature continues to report substantial variability in accuracy [18–20]. Furthermore, in vivo clinical studies evaluating digital implant impressions obtained with IOS and assessing the fit of the resulting restorations remain limited [21, 22]. In addition to the impression technique, factors such as the implant number and deviation between them, the manufacturing method, and the material of the restoration have also been shown to influence the achievement of a passive fit [23, 24].

When an implant-supported restoration fails to achieve a passive fit, it can be sectioned and reconnected directly in the patient’s mouth. To preserve a fully digital workflow, reverse scan bodies may be attached to the verification stent or the temporary and scanned extraorally. The principle of reverse scan bodies is to digitally capture implant-supported provisional restorations or frameworks by connecting scan bodies to the prosthesis outside the mouth, allowing precise transfer of implant positions and the corresponding prosthetic geometry into the CAD environment [25]. These data can then be used to fabricate a new restoration. Although reverse scan bodies can be used in two-implant situations, their clinical relevance is expected to be greater in full-arch All-on-X and other multi-implant restorations, where passive fit is more difficult to achieve. A similar concept, avoiding intraoral sectioning and reconnection, has previously been applied during the initial fabrication of definitive implant restorations, provided that a passively fitting interim prosthesis was available [26, 27]. This method evolved from a semi-digital laboratory protocol in which conventional implant impressions are scanned to record implant locations, eliminating the need for a physical implant master model. Despite several reports describing the use of reverse scan bodies in the literature, no prospective clinical feasibility evaluations have yet assessed the accuracy of this approach in the edentulous mandible using a fully digital workflow [28, 29].

The objective of this single-patient clinical evaluation was to assess both the clinical feasibility and accuracy of a completely digital approach for creating implant-supported restorations in one edentulous mandible restored with two interforaminal implants. This study focused on intraoral scanning for initial data capture and on using reverse scan bodies to record implant positions after intraoral re-splinting. The number of measurements was determined according to previous studies [30, 31], which was considered sufficient to assess variability. The findings were intended to provide preliminary technical and clinical observations rather than generalizable evidence for edentulous arches or multi-implant full-arch restorations.

## Methods

### Study participant

**Fig. 1:**
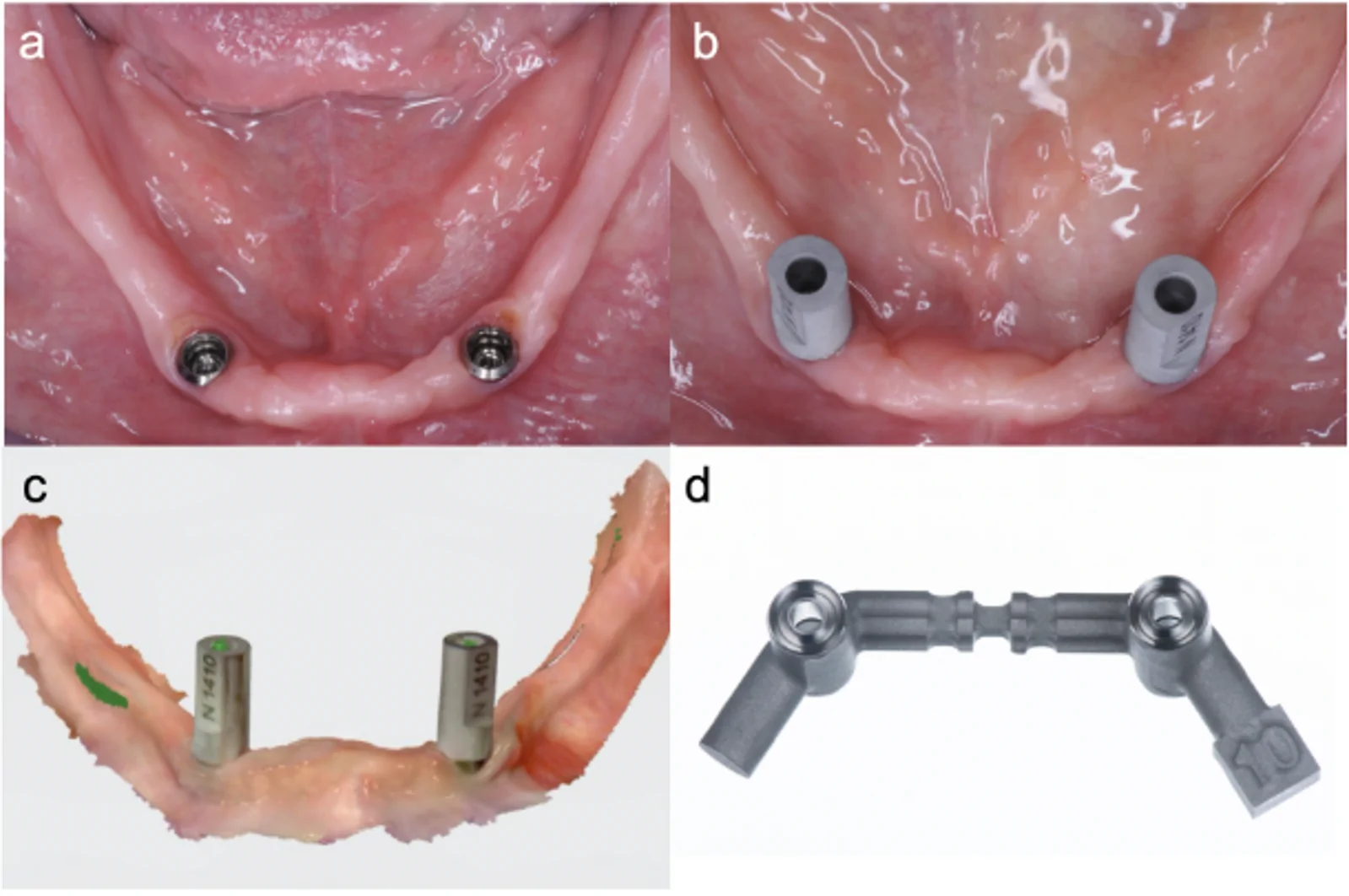
(**a**) Intraoral clinical situation of the edentulous mandible with two tissue-level implants positioned in the canine region; (**b**) scan bodies attached to the implants; (**c**) intraoral scan; (**d**) CAD-CAM manufactured titanium verification bar (VB) with model-free cemented titanium bases

A female patient (53 years) with a fully edentulous mandible was included in this study at the Department of Reconstructive Dentistry, University Center for Dental Medicine, Basel, Switzerland. Written informed consent was obtained prior to participation. Implant placement was planned using computer-assisted virtual planning with coDiagnostiX software (Dental Wings GmbH, Chemnitz, Germany). According to the digital treatment plan, two tissue-level implants (Institut Straumann AG, Basel, Switzerland) were placed in the mandible (both 3.3 × 10 mm in the canine region, i.e. areas 33 and 43 according to FDI notation; Fig. 1a). Ethical responsibility for the study protocol was clarified by the Ethics Committee Northwest and Central Switzerland (EKNZ-ID 2021 − 00249), and all procedures were conducted in accordance with the Declaration of Helsinki and applicable national regulations.

### Digital workflow

**Fig. 2:**
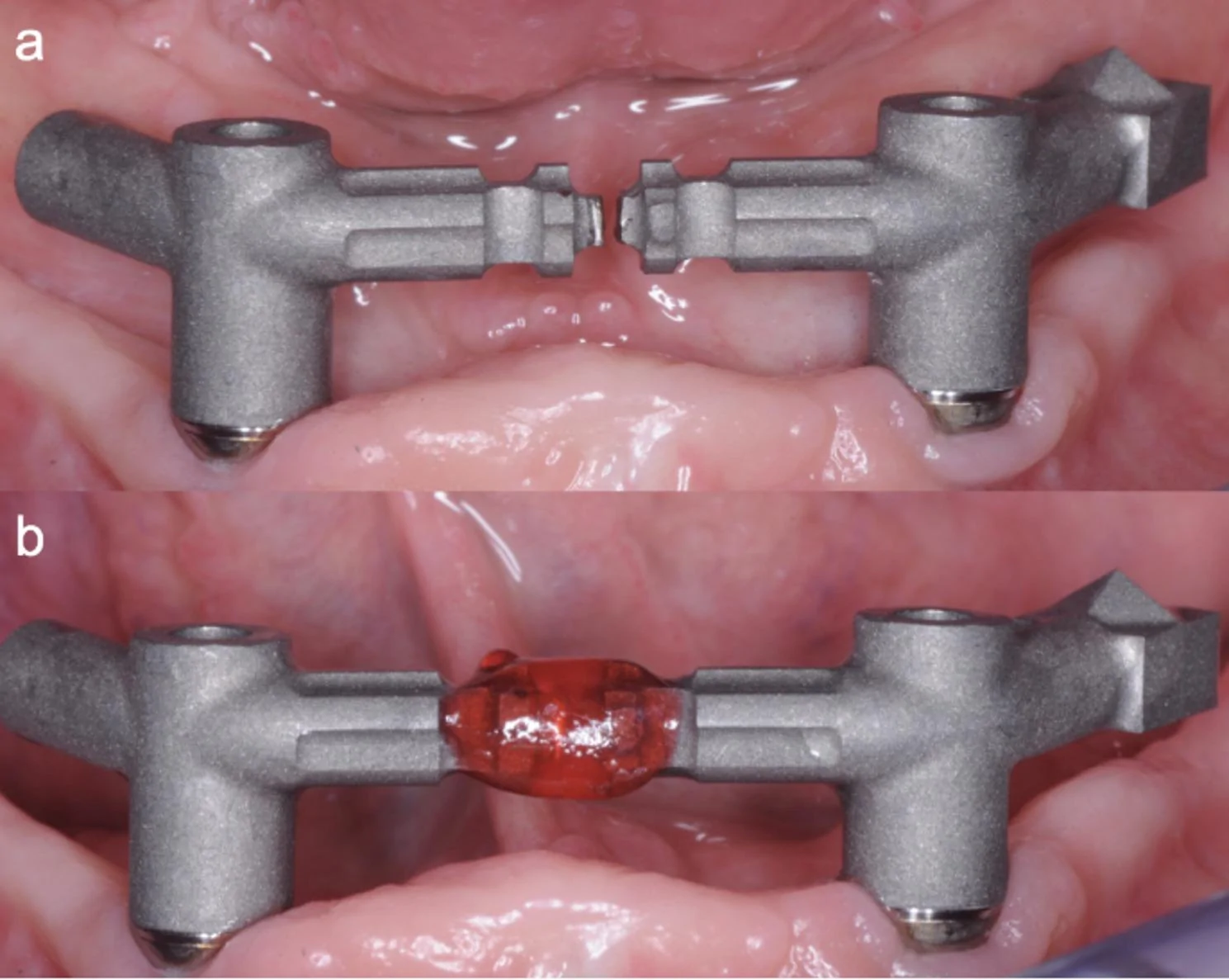
(**a**) Sectioned verification bar (VB) and (**b**) intraorally re-splinted verification bar (SVB) using light-polymerizing acrylic resin

Scan bodies were mounted onto the implants and remained in situ throughout the entire digital impression process (Fig. 1b). A lip and cheek retractor (OptraGate, Ivoclar Vivadent AG, Schaan, Liechtenstein) was used to keep the soft tissues in the edentulous areas as stable as possible. Intraoral scanning was performed by an experienced operator (A. K.-G.) using a calibrated TRIOS 4 scanner (3Shape, Copenhagen, Denmark; software version 21.2.0), under standardized ambient lighting conditions and in accordance with the manufacturer’s recommendations (Fig. 1c). The scanning pattern began on the occlusal surface in the fourth quadrant, moving towards the contralateral side. The scanning process continued along the lingual side and was concluded capturing the buccal surface. A total of ten intraoral scans with fully captured scan bodies and residual mandibular ridge were obtained (IOS group; *n* = 10). Based on each intraoral scan, a titanium verification bar (VB) was designed and manufactured using a fully digital CAD/CAM workflow (*n* = 10; Exocad Dental CAD 3.1 Rijeka 8349, exocad GmbH, Darmstadt, Germany; PowerMill 2016 R2, Autodesk, San Francisco, USA; Fig. 1d). Subsequently, titanium bases (RN Variobase for bridge/bar cylindrical, 5 × 3.5 mm; Institut Straumann AG, Basel, Switzerland) were adhesively cemented to the VB using a dual-curing resin cement (Multilink Hybrid Abutment, Ivoclar Vivadent, Schaan, Liechtenstein), without the fabrication of a physical model. The passive fit of each numbered VB was evaluated clinically in random order by visual inspection using a dental probe, tactile assessment, and application of the Sheffield test with manual light tightening of a single screw on the most distal implant and evaluation of passive fit at the opposite site [7]. The evaluator (A. K.-G.), who had more than 10 years of clinical experience, was calibrated to ensure standardized assessment of clinical passive fit. For digital analysis, the VBs were then scanned using an optical laboratory scanner (inEos X5, Dentsply Sirona, Bensheim, Germany; inLab software version 22.1.1.276516) with reverse scan bodies attached. Following the initial evaluation, the VBs were sectioned and intraorally re-splinted using a light-polymerizing acrylic resin (Primopattern LC Gel, primotec, Bad Homburg, Germany; Fig. 2). The resulting splinted verification bars (SVBs; *n* = 10), equipped with reverse scan bodies, were subsequently digitized using the same laboratory scanner. Based on these data, new verification bars (NVBs; *n* = 10) with integrated titanium bases were milled. The passive fit of the NVBs was again assessed clinically. For further digital analysis, the NVBs, with reverse scan bodies attached, were scanned using the laboratory scanner.

### Data analysis

All datasets obtained from the IOS, VB, SVB, and NVB groups were exported in standard tessellation language (STL) format and imported into metrology software (Geomagic Control X, version 2018.1.2; 3D Systems, Rock Hill, SC, USA) for further evaluation. Following an established protocol described previously [32], a reference coordinate system was generated for each scan by defining a vertical axis and a corresponding reference plane at the coronal surface of the scan bodies. Based on these reference geometries, spatial relationships between the two scan bodies were quantified. For each dataset, linear distances and angular deviations were calculated. Linear measurements were derived from the intersection points of the vertical axes with the reference plane, whereas angular measurements were computed from the orientation of the respective vertical axes of the two scan bodies.

### Statistical analysis

Because the study design was based on repeated scans, no formal sample size calculation for independent observations was performed. The number of measurements was based on previous studies [30, 31] and was considered sufficient to evaluate measurement variability. Therefore, the statistical analyses should be interpreted as descriptive and exploratory and were not intended for formal hypothesis testing.

All statistical evaluations were carried out using the Python programming environment (version 3.12; Python Software Foundation, Wilmington, DE, USA). Because all measurements were obtained from repeated scans and restorations in a single patient, the statistical analysis was primarily descriptive and exploratory. Data distribution was assessed using the Shapiro–Wilk test, and variance homogeneity was examined with Levene’s test. Linear and angular measurements from the IOS, VB, SVB, and NVB groups were compared using Welch’s analysis of variance (ANOVA) followed by Games-Howell post hoc tests when applicable. To control for type I error arising from multiple testing, p-values were adjusted using the Holm–Bonferroni correction. A threshold of α = 0.05 was used for exploratory comparisons, but p-values should not be used to draw definitive conclusions.

## Results

### Clinical examination

**Fig. 3:**
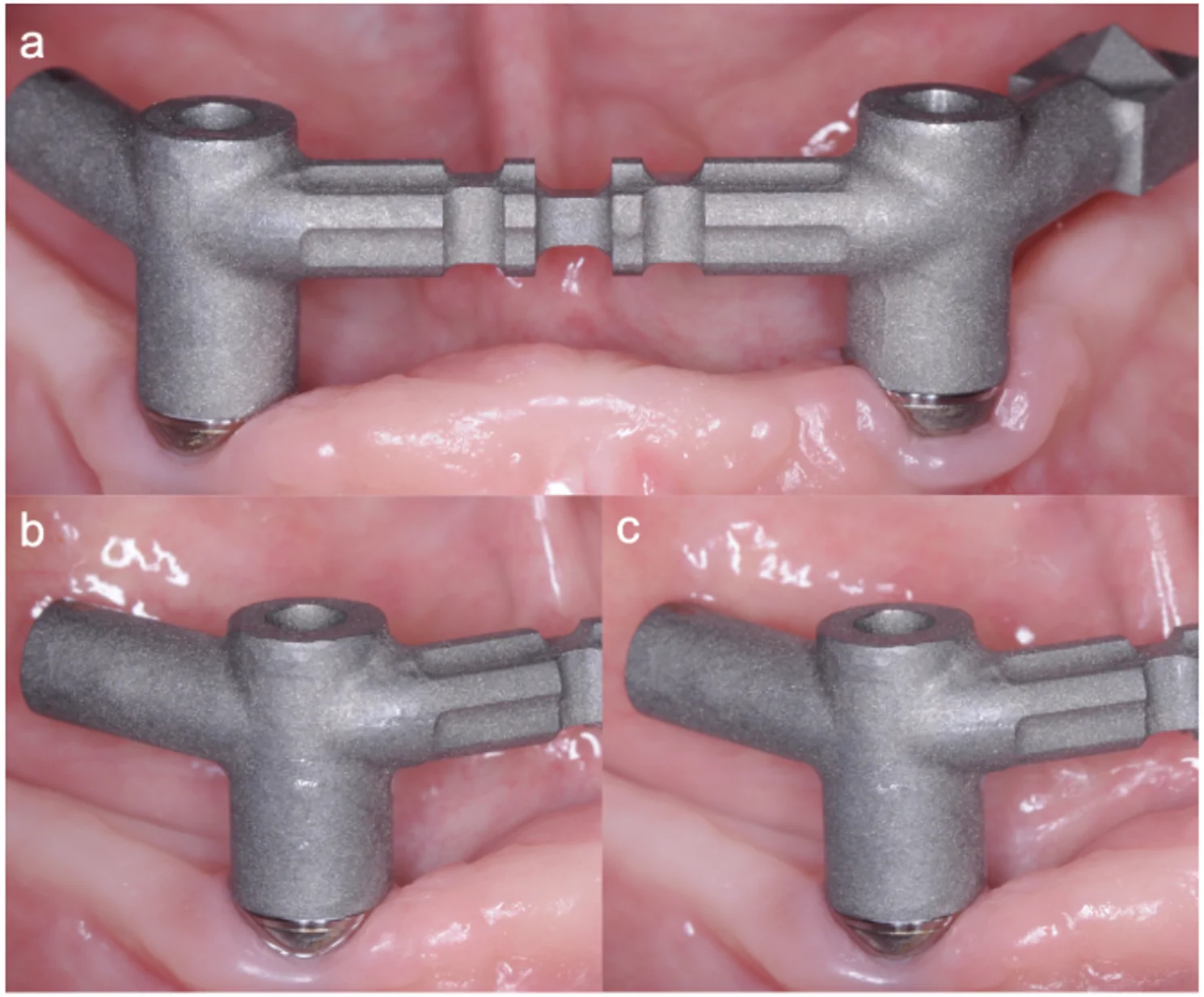
(**a**) Representative clinical try-in of a verification bar; (**b**) passive fit assessment using the Sheffield test with the screw tightened at the right implant site (region 43); (**c**) passive fit evaluation after removal of the screw at the right implant, while the screw at the left implant site (region 33) remained tightened

The clinical assessment revealed passive fit during visual and tactile inspection in combination with the Sheffield test in all ten VBs (Fig. 3). Clinical evaluation of the NVBs, including the Sheffield test, demonstrated passive fit on both implants in all ten NVBs.

### Linear/angular deviations

#### Linear deviations

**Fig. 4:**
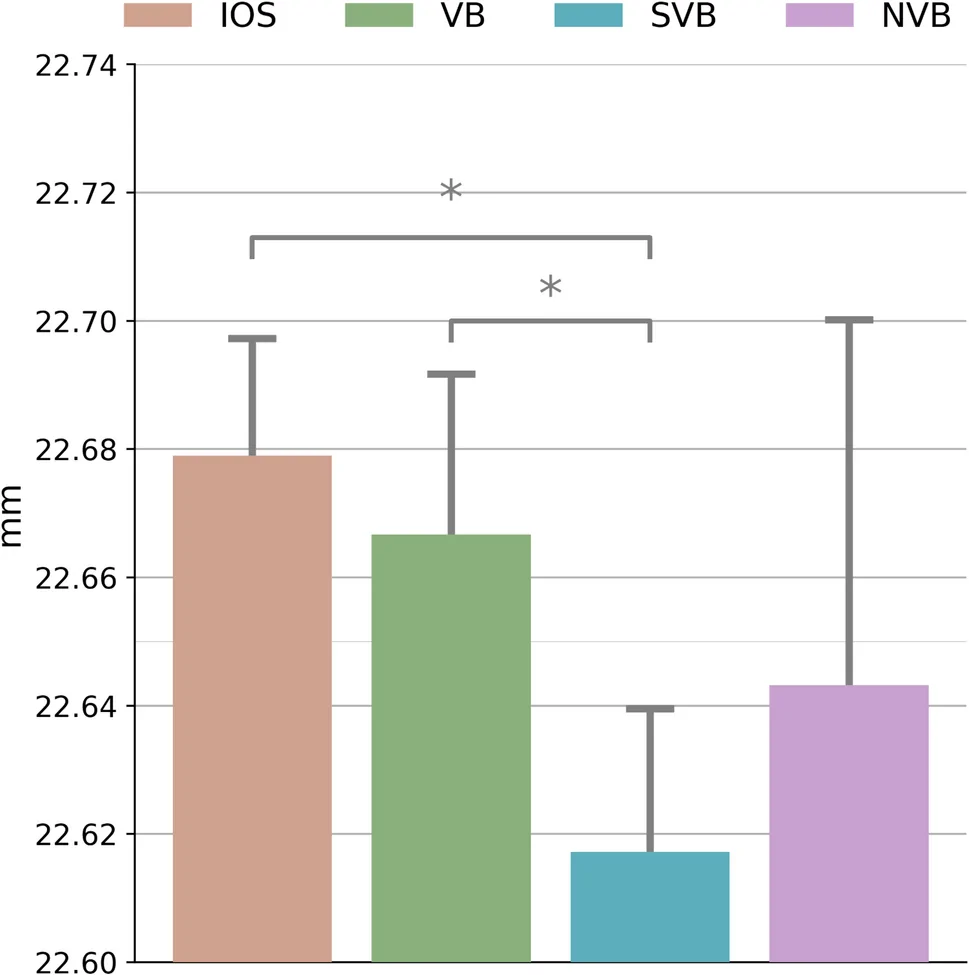
Implant distance measurements recorded intraorally using an intraoral scanner (IOS) and extraorally using an optical laboratory scanner for the VB, SVB, and NVB after attachment of reverse scan bodies. Asterisk (*) indicates significant difference, *p* < 0.05

The inter-implant distances and linear deviations for the different datasets (IOS, VB, SVB, and NVB) are summarized in Table 1; Fig. 4. In the comparison between IOS and VB, a small mean linear deviation of 12 μm was observed between the two implants, with no statistically significant difference between the two datasets (*p* = 0.23). The highest inter-implant deviation was 43 μm. When comparing VB and SVB, the mean deviation increased to 50 μm, and this difference was statistically significant (*p* = 0.001). The maximum deviation measured here was 88 μm. In contrast, the comparison between SVB and NVB revealed a mean deviation of 26 μm, which was not statistically significant (*p* = 0.20). One outlier > 100 μm here was observed (126 μm). The highest mean deviation was measured between IOS and SVB (62 μm), which was statistically significant (*p* < 0.001).

**Table 1:** Inter-implant distances (mm) and corresponding linear deviations (µm) calculated between IOS and VB, VB and SVB, and SVB and NVB. IOS = intraoral scan; VB = verification bar; SVB = re-splinted verification bar; NVB = new verification bar

Comparison (Group 1-Grroup 2)	Group 1 Mean (± SD), mm	Group 2 Mean (± SD), mm	Mean diff, µm	*p*-value
IOS-VB	22.679 (0.018)	22.667 (0.025)	12	0.23
VB-SVB	22.667 (0.025)	22.617 (0.022)	50	0.001
SVB-NVB	22.643 (0.057)	22.617 (0.022)	26	0.20
IOS-SVB	22.679 (0.018)	22.617 (0.022)	62	< 0.001
IOS-NVB	22.679 (0.018)	22.643 (0.057)	36	0.29
VB-NVB	22.667 (0.025)	22.643 (0.057)	24	0.64

#### Angular deviations

**Fig. 5:**
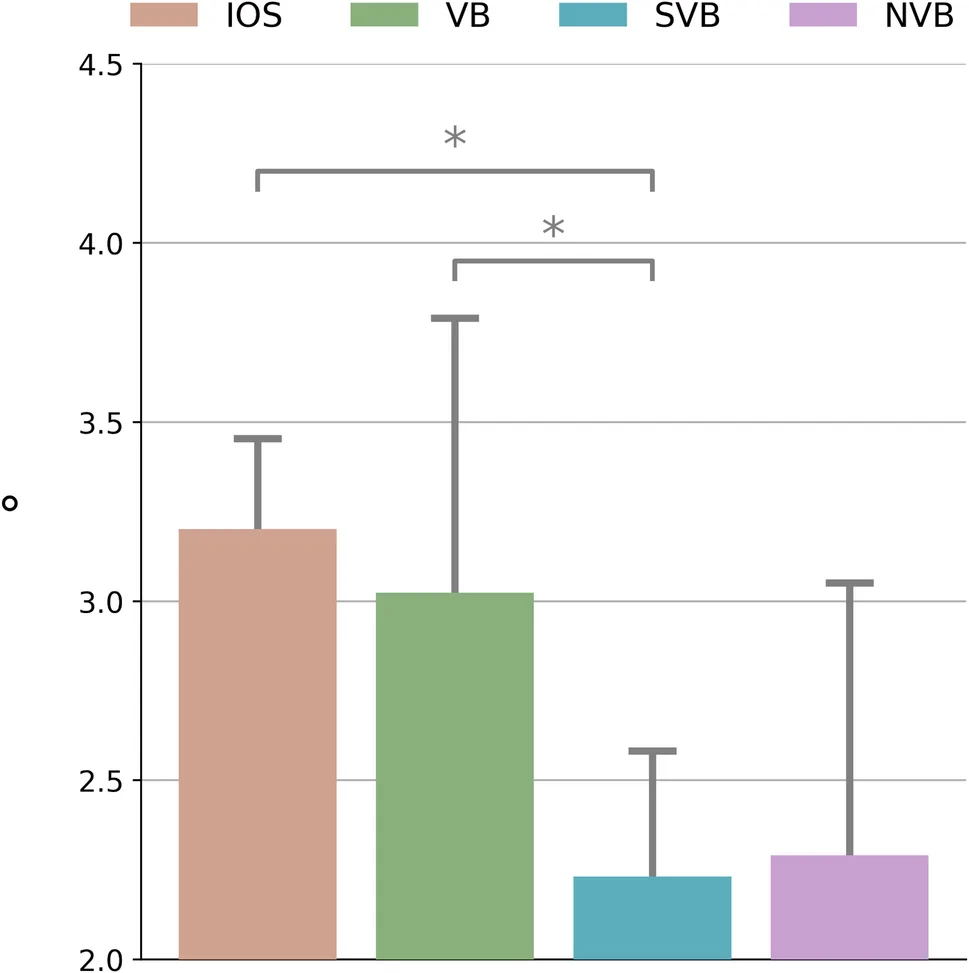
Measured angles between implants using IOS for the intraoral situation and an optical laboratory scanner for VB, SVB, and NVB with attached reverse scan bodies. Asterisk (*) indicates significant difference, *p* < 0.05

The inter-implant angles and angular deviations between the two implants across different datasets (IOS, VB, SVB, NVB) are shown in Table 2; Fig. 5. In the IOS-VB comparison, the mean angular deviation was 0.18° (*p* = 0.50). Out of the ten measured inter-implant angles, six exceeded 0.4°, with the highest angle deviation reaching 0.99°. In the VB-SVB comparison, the mean angular deviation increased to 0.79° (*p* = 0.01). Seven measurements exceeded the threshold of 0.4° and reached a maximum of 1.91°. In the SVB-NVB comparison, the mean angular deviation between the two implants was minimal (0.06°; *p* = 0.82). However, four measurements ranged from 0.53° to 1.92°.

**Table 2:** Inter-implant angles (°) and corresponding angular deviations (°) calculated between IOS and VB, VB and SVB, and SVB and NVB. IOS = intraoral scan; VB = verification bar; SVB = re-splinted verification bar; NVB = new verification bar

Comparison (Group 1-Group 2)	Group 1 Mean (± SD), °	Group 2 Mean (± SD), °	Mean diff, °	*p*-value
IOS-VB	3.2 (0.25)	3.02 (0.77)	0.18	0.50
VB-SVB	3.02 (0.77)	2.23 (0.35)	0.79	0.01
SVB-NVB	2.29 (0.76)	2.23 (0.35)	0.06	0.82
IOS-SVB	3.2 (0.25)	2.23 (0.35)	0.97	< 0.001
IOS-NVB	3.2 (0.25)	2.29 (0.76)	0.91	0.02
VB-NVB	3.02 (0.77)	2.29 (0.76)	0.73	0.17

## Discussion

This single-patient prospective clinical feasibility evaluation assessed the accuracy of digital impressions of one edentulous mandible with two implants placed in the canine regions by fabricating and clinically evaluating titanium VBs. All ten VBs demonstrated clinically passive fit upon evaluation, including the Sheffield test. The Ti-bases of the VBs were cemented without a model; therefore, the study additionally investigated whether measurable differences could be identified in comparison with NVBs after intraoral splinting and reconnection, in which the Ti-bases were already integrated. Although all ten VBs were clinically assessed as passive, the potential measurable effect of intraoral splinting was further evaluated.

Numerous studies [15, 33–35] have similarly compared deviations between different datasets regarding the accuracy of digital implant impressions in the edentulous jaw. A systematic review evaluating various digital technologies for implant impressions in the edentulous jaw was able to include only studies that did not involve clinical try-in of frameworks [36]. Only one study assessed frameworks on a model produced from a conventional impression as a control [37]. The authors therefore concluded that future studies should also investigate the clinical fit of full-arch implant-supported prostheses [35]. A major strength of the present investigation is that the observed deviations were not evaluated solely against theoretical thresholds; instead, rigid titanium VBs were fabricated and clinically tested for passive fit.

In a comparable clinical study by Andriessen et al. [14], 25 patients with two mandibular implants were evaluated. Of the 21 scans that could be analyzed, only five demonstrated scan distance errors < 100 μm when compared with a clinically validated reference, while four additional scans were excluded due to stitching errors. Only three of the 21 scans exhibited angulation errors < 0.4°. Owing to the high number of large distance and angular errors, the authors concluded that an adequate passive fit of the restorations could not be achieved. These findings are not consistent with the results of the present study. When comparing the inter-implant distance error between IOS and VB, a mean deviation of only 12 μm was observed, with a maximum measured deviation of 43 μm. In addition, the mean angular error was limited to 0.18°. The clinically observed passive fit confirmed that these minimal linear and angular deviations did not result in any clinically relevant misfit. Other, more recent in vivo studies have reported linear deviations of varying magnitude for digital impressions compared with conventional references, with mean values of up to 162 μm [15, 35, 36].

The favorable results observed in the present study should be interpreted in light of the specific hardware, software, and clinical condition. The use of a contemporary IOS system may have contributed to the low deviations, as significant differences in accuracy between systems have been reported over time [38]. However, the reliance on a single IOS, one scan body design, one implant system, one CAD/CAM workflow including one metrology software, and one laboratory scanner is an important limitation because proprietary stitching algorithms and scanner-specific acquisition strategies may influence accuracy. In addition to operator-related factors, such as ambient lighting conditions and operator experience, patient-related factors may also have influenced scan quality [39, 40]. In this context, inter-implant distance has been shown to affect accuracy, with larger distances correlating with increased inaccuracies. The relatively short inter-implant distance of 22 mm in the present study may therefore have contributed to the high level of accuracy observed. Furthermore, it is well established that increased implant angulation can lead to greater scanning inaccuracies [24, 41]. However, this effect is generally reported in the literature only for implant angulations exceeding 30° [42, 43]. Consequently, the nearly parallel alignment of the two implants in the present study (2–3° angulation) likely facilitated accurate intraoral data acquisition. An additional advantage in this case may have been the presence of a characteristic alveolar ridge despite present bone loss, which supported accurate image stitching during the scanning process. A positive correlation between the number of implants and deviations in the edentulous maxilla has previously been observed in a cohort of 16 patients [15]. The patients received between four and six implants, and an increase in deviations was documented with a higher number of implants, although this correlation did not reach statistical significance. Therefore, the present findings from a short-span, two-implant mandibular situation cannot be extrapolated to full-arch multi-implant restorations, larger inter-implant distances, maxillary edentulous arches, severely resorbed ridges, or cases with greater implant divergence.

By separating and intraorally reconnecting the VBs, statistically significant linear differences compared with the SVBs were detected; nevertheless, these differences did not compromise the clinically verified passive fit of the VBs. Despite being measurable and statistically significant, these differences must be considered small, amounting to 50 μm compared with the VB and 62 μm compared with the IOS. These findings support the widely accepted assumption that linear deviations of < 100 μm do not result in a clinically relevant misfit [14]. In contrast, angular deviations between the two implants exceeded the widely accepted threshold of 0.4° (0.79° between SVB and VB and 0.97° between SVB and IOS) [14, 30]. Nevertheless, a passive fit of the VBs was achieved in this single-patient evaluation indicating that the observed angular deviations did not result in a detectable clinical misfit under the specific conditions investigated. This observation should be interpreted as preliminary support for further studies designed to correlate angular deviations with objective measures of fit and clinical outcomes. In line with these findings, Carneiro Pereira reported in an in-vivo study using the TRIOS system that minimal linear deviations (< 5 μm) were not necessarily associated with low angular deviations [34].

The measurable differences between SVB and NVB were marginal in both linear and angular terms (26 μm and 0.06°, respectively). In conjunction with the clinically verified passive fit of all ten NVBs, these findings indicate that the protocol using reverse scan bodies and extraoral digitization may be clinically applied when a non-passive fit of the restoration is observed after splinting and reconnection. While the present evaluation involved only two interforaminal implants, the reverse scan body concept may be particularly relevant for All-on-X and other full-arch multi-implant reconstructions, where passive fit is more challenging than in two-implant overdenture-related workflows. A prerequisite for this approach is a highly accurate manufacturing process of the restoration to avoid the introduction of additional inaccuracies [44]. Other authors have also described that this protocol may be used for the fabrication of a definitive implant-supported prosthesis, provided that an adequately fitting interim prosthesis is available [26–28]. This approach may be particularly advantageous in the mandible, where the clinical situation, unlike in the present study, may be complicated by advanced mandibular resorption, flabby mucosal tissues, or excessive saliva [27]. However, the influence of the edentulous arch (maxillary versus mandibular) on the accuracy of implant impressions remains controversial, as the literature reports partially contradictory findings [33, 41, 45, 46]. For a definitive evaluation, the interim prosthesis should be fabricated with a rigid metal framework to ensure true passive fit, as acrylic materials may deform during verification and falsely suggest passivity.

The present investigation is subject to several important limitations. Its single-patient design represents only one distinct clinical constellation with respect to implant system, implant angulation, inter-implant distance and other anatomical characteristics [47]. Repeated scans and repeated bars provide information on this workflow in one clinical situation, but they do not replace biological variation across independent patients. In addition, the absence of a true physical gold-standard reference, such as a conventional splinted open-tray elastomeric impression and validated master cast, limits the ability to determine absolute trueness. However, such a model would also represent an additional reference rather than an absolute intraoral “true” situation, since errors may occur during impression taking, material setting, implant analog connection, and cast fabrication. The study protocol also incorporated only a single IOS, one scan body design, one restoration fabrication workflow, one metrology software, and one laboratory scanner, thereby limiting the external validity and transferability of the findings [24, 38, 48]. Furthermore, passive fit was assessed clinically by visual inspection, tactile assessment, and the Sheffield test; although these methods are widely used clinically, they remain partly subjective and do not quantify microscopic gaps or internal non-passive stresses within the framework. Therefore, the results should be interpreted as preliminary feasibility data and should not be generalized to full-arch multi-implant restorations, maxillary edentulous arches, severely resorbed ridges, longer implant spans, or cases with greater implant divergence. To confirm and extend the present results, future randomized clinical trials with larger and more diverse patient populations are required, encompassing a wider range of anatomical situations, implant configurations, and digital workflows. Particular attention should be directed toward the evaluation of horizontal scan bodies with defined geometries, as emerging evidence suggests that they may enhance the accuracy of digital impressions in fully edentulous arches by reducing the risk of image stitching errors [49, 50]. This concept is consistent with alternative strategies aimed at improving scan stability, such as splinting vertical scan bodies or incorporating soft tissue landmarks into the scanning process [20, 51]. However, the frequent implementation of horizontal scan bodies within proprietary closed software ecosystems may restrict broader applicability. In addition, photogrammetry has shown considerable potential for the highly accurate acquisition of implant positions in edentulous jaws, demonstrating excellent precision for the fabrication of implant-supported restorations [52–54]. Despite these advantages, widespread clinical adoption may be constrained by high equipment costs, limited accessibility, and the requirement for specialized training. Furthermore, photogrammetry is inherently restricted to the registration of implant positions and must be combined with intraoral scanning to capture peri-implant soft tissue morphology.

## Conclusions

Within the limitations of this single-patient clinical evaluation, intraoral scanning allowed the digital fabrication of passively fitting implant-supported restorations for one favorable edentulous mandibular case restored with two nearly parallel interforaminal implants. Measurable linear and angular deviations did not compromise clinically assessed passivity in this specific setting. Furthermore, the described approach, combining separation, intraoral re-splinting, and extraoral digitization with attached reverse scan bodies, may represent a clinically viable option when misfit is suspected in a fully digital workflow, particularly as a corrective strategy that may be relevant for more complex full-arch situations. These preliminary findings cannot be extrapolated to multi-implant full-arch restorations, maxillary edentulous arches, severely resorbed ridges, or greater implant divergence without further evidence. Prospective clinical studies with larger cohorts and objective fit assessments are required to validate these findings.

## Data Availability

The datasets used and/or analyzed during the current study available from the corresponding author on reasonable request.

## Abbreviations

IOS: Intraoral scan
VB: Verification bar
SVB: Splinted verification bar
NVB: New verification bar
STL: Standard tessellation language
